# Design and Realization of Ni Clusters in MoS_2_@Ni/RGO Catalysts for Alkaline Efficient Hydrogen Evolution Reaction

**DOI:** 10.3390/molecules28186658

**Published:** 2023-09-16

**Authors:** Haifeng Xu, Nannan Liang, Zhi Bai, Bo Yang, Dongmeng Chen, Huaibao Tang

**Affiliations:** 1 School of Information Engineering, Suzhou University, Suzhou 234000, China; 2 School of Mechanics and Materials, Hohai University, Nanjing 211100, China; 3 School of Mechanical and Electronic Engineering, Suzhou University, Suzhou 234000, China; 4 Anhui Province Key Laboratory of Pollutant Sensitive Materials and Environmental Remediation, School of Physics and Electronic Information, Huaibei Normal University, Huaibei 235000, China; 5 College of Science, China University of Petroleum, Qingdao 266580, China; 6 School of Materials Science and Engineering, Anhui University, Hefei 230601, China

**Keywords:** nickel clusters, MoS_2_@Ni/RGO catalyst, HER

## Abstract

Due to their almost zero relative hydrogen atom adsorption-free energy, MoS_2_-based materials have received substantial study. However, their poor electronic conductivity and limited number of catalytic active sites hinder their widespread use in hydrogen evolution reactions. On the other hand, metal clusters offer numerous active sites. In this study, by loading Ni metal clusters on MoS_2_ and combining them with the better electrical conductivity of graphene, the overpotential of the hydrogen evolution reaction was reduced from 165 mV to 92 mV at 10 mA·cm^−2^. This demonstrates that a successful method for effectively designing water decomposition is the use of synergistic interactions resulting from interfacial electron transfer between MoS_2_ and Ni metal clusters.

## 1. Introduction

Since the supply of fossil fuels is quickly running down and governments place a high priority on protecting the environment, green and clean energy sources are becoming increasingly necessary [1]. Hydrogen, with its high calorific value, highly efficient thermal conductivity, and non-toxicity, has many readily apparent advantages, including easy transportation, efficient energy utilization, and non-toxic emissions. Moreover, when hydrogen is used as an energy carrier compared to conventional fossil fuels, it only emits water and no other harmful compounds, which aligns with the needs of green energy growth. However, the current main source of hydrogen energy relies on conventional fossil fuels for production, which harms the environment and makes its future development prospects unsustainable. The future development of electricity is moving towards green hydrogen, which can be manufactured by electrolyzing water and causes less damage to the environment [2,3]. Recently, noble metal catalysts, such as Pt, Ir, and others, have been found to be more efficient HER catalysts due to their low resistance coefficients and high activity. However, their limited accessibility hinders their frequent use in electrolytic water [4,5]. The HER reaction is a two-electron-transfer electrochemical reaction. The development of catalysts with high efficiency and stable alkalinity is of great significance for the production of green hydrogen. This is because in an acidic environment the number of protons is higher than in an alkaline environment, and extra energy is required to break the covalent H-O-H bond to form protons in an alkaline environment [6,7].

The most widely recognized instance of electrocatalytic synergy in current research is the alloy of Ni and Mo, which results in HER efficiency equal to Pt/C [8,9]. Due to its outstanding catalytic activity, it has been used as a benchmark catalyst for HER of non-precious metals. Increasing the electrical conductivity of the catalyst itself can effectively promote the transmission of current on the catalyst surface and thereby improve the efficiency of the electrocatalytic hydrogen precipitation reaction, in addition to the synergistic effect between the bimetallic Ni-Co and Ni-Fe [9,10]. For example, the conductivity of the catalyst can be significantly increased by mixing conductive elements, such as carbon nanotubes and graphene, with the catalytic material. It is crucial to scientifically construct and modify the catalyst’s electrical structure to further improve its electrocatalytic performance. This is mainly due to the fact that the electronic structures of catalysts have a direct impact on their catalytic activity and selectivity, making it possible to regulate and optimize catalytic events by changing the electronic structure. One example is the use of surface modification techniques to alter the electrical structure through molecule adsorption on the catalyst surface. Another example is the addition of auxiliary materials, such as transition metal oxides or sulfides, to the catalyst surface, which allows control over the surface’s electronic state, adsorption active sites, and charge distribution. A faster transition of in situ charge, on the other hand, refers to altering the electron transport and charge distribution of the reactants at the catalytic interface to speed up the catalytic reaction. The concept has two fundamental components: in situ charge and charge transfer. In situ charge refers to the charge state of reactant molecules or ions on the catalyst surface or catalytic interface. By altering the electron distribution and potential energy of the in situ charge, a faster transition is made possible. Charge transfer, on the other hand, refers to the transmission of electrons from one substance to another. In electrocatalytic hydrogenolysis, electrons can be taken from the catalyst’s surface or given to the reactants, which causes them to undergo an electron transfer process and speeds up the catalytic reaction. These synergistic effects have been shown to be productive [11,12,13,14].

One of the non-precious metal catalysts, MoS_2_, has been suggested to have great potential as a base agent for catalysis due to its nearly negligible relative hydrogen atom adsorption-free energy [15]. However, its poor electrical conductivity and limited number of active sites are significant limitations. Heteroatom doping, defect engineering, and interfacial engineering are the three primary approaches used to address these issues [16,17,18]. Interfacial engineering with hybridization could be utilized to modify the hydrogen binding on MoS_2_ edges [19]. According to an electronic structure study, the downward shift of the S-atom’s p-state and the adsorbed state of the hydrogen atom at the MoS_2_ edge resulted in an increase in the filling of the antibonding state and a weakening of the hydrogen binding [20]. Metal clusters function well as catalysts in alkaline hydrogen precipitation processes due to their high specific surface areas and abundance of surface active sites. This is because metal clusters provide sites for the adsorption and activation of hydrogen ions in water molecules. They can assist in the transfer of electrons by hydrogen ions and reduce the activation energy of the reaction, thereby accelerating the hydrogen precipitation process. However, there has not been sufficient attention given to the interfacial electron transfer resulting from doping metal clusters based on interfacial engineering. To further enhance catalytic efficiency, it is practical to have a thorough understanding of the synergistic mechanisms underlying them.

After conducting electrochemical and characterization tests, we discovered that Ni metal clusters can enhance the catalytic activity of HER under various current density conditions. This was achieved by co-precipitating Ni metal clusters with MoS_2_, which was synthesized using the hydrothermal method [21]. The high hydrogen affinity of metallic Ni clusters hinders their potential as HER catalyst candidates due to unfavorable hydrogen recombination [22,23,24]. However, the combination of heteroatomic nickel doping and interfacial engineering yielded unexpected results. This suggests that water dissociation can be effectively strengthened and that catalytic efficiency can be further increased through the redistribution of interfacial electron transfer based on synergistic interactions.

## 2. Results and Discussion

### 2.1. Morphologies and Structure of MoS_2_@Ni/RGO

**Scheme 1:**
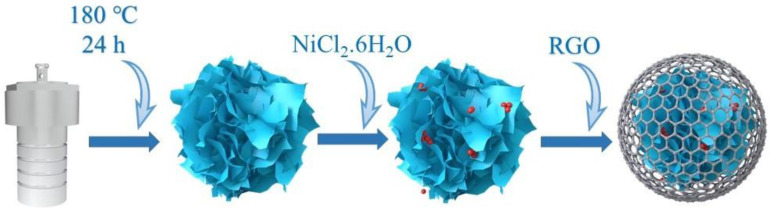
Synthesis process of MoS_2_@Ni/RGO.

**Figure 1:**
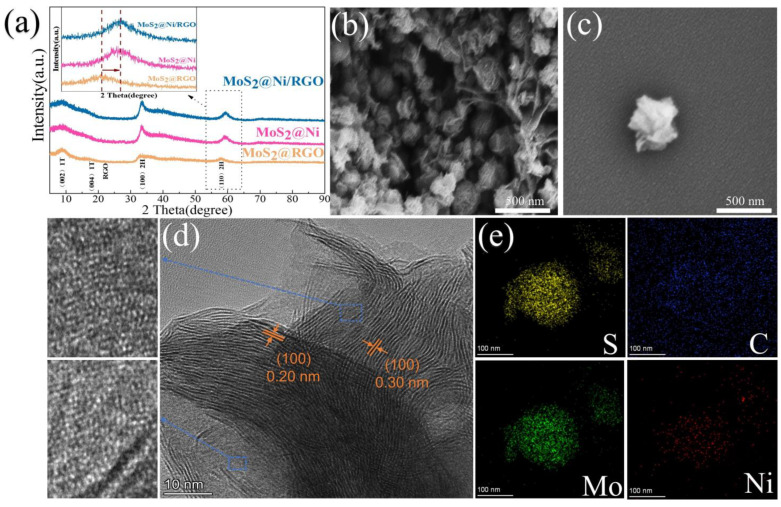
(**a**) XRD patterns of MoS_2_@RGO, MoS_2_@Ni, and MoS_2_@Ni/RGO. (**b**,**c**) SEM images of MoS_2_@Ni/RGO. (**d**) HRTEM images of MoS_2_@Ni/RGO. (Blue boxes guide the eye to show the presence of Ni clusters on MoS_2_, and white highlights the lattice stripes corresponding to MoS_2_). (**e**) Corresponding EDS elemental mappings of Ni, C, Mo, and S, respectively.

MoS_2_@Ni/RGO composites that resemble flowers were synthesized, as shown in Scheme 1. After pyrolysis in a mixture of hydrogen (5%) and nitrogen (95%), the flower-like MoS_2_ prepared by the hydrothermal method retained its original flower-like morphology (Figure 1b,c). This demonstrates that the MoS_2_ morphology was effectively preserved even after the addition of nickel clusters and graphene to the 1T-2H-MoS_2_ particles. The XRD spectra of the 1T-2H-MoS_2_ complex show two peaks at 32.6° and 58.2°, which correspond to the (100) and (110) crystal faces of 2H MoS_2_, respectively (Figure 1a) [25]. Two more full peaks could have been found at 9.63° and 19.7°, respectively, which somewhat correspond to the (002) and (004) planes of the magnetic 1T MoS_2_ [26]. Since the nickel diffraction peaks were not apparent in Figure 1a, it was confirmed that the nickel content of the material is very low. The (110) face of the 2H MoS_2_ crystal structure was moved at the 58.2° location in the MoS_2_ material after loading nickel clusters (MoS_2_@Ni/RGO) as compared to the MoS_2_ material without nickel clusters, according to the magnified processed XRD patterns. The loaded nickel clusters’ compressive strain action was responsible for this event. The MoS_2_ material’s crystal structure was altered by the loading of nickel clusters when weighed compared to the unloaded nickel clusters, moving the XRD peak at 58.2° in the (110) face. Size matching, angular effect, and surface effect are all capable of being utilized to explain this compressive strain effect. The nickel-loaded clusters are permitted to be inserted in the MoS_2_ lattice due to their size compatibility, which results in compression of the lattice around it. On top of that, the presence of clusters alters the angle at which nearby atoms face one another, further distorting the lattice. The shift of the XRD peak at 58.2° can be linked to the compressive strain effect brought about by the loaded nickel clusters by comparing the MoS_2_ materials with different loaded nickel clusters. In addition, the attachment of clusters on the MoS_2_ surface also leads to the distortion and compression of the surface atoms, which results in a compressive strain effect inside the crystal [27]. Ultra-high resolution scanning electron microscopy (SEM) shows the morphology of MoS_2_@Ni/RGO as shown in Figure 1b. MoS_2_@Ni showed an overall spherical pattern beneath the coating of graphene, which is thought to be due to excellent electrical conductivity and is also thought to hasten the material’s kinetic reaction process [28,29]. The microstructure has been further explained using HRTEM. While the nickel clusters in the blue-line box are equally spread across the MoS_2_ surface, the lattice spacing of 0.30 nm in Figure 1d corresponds to the (100) face of 1T-phase MoS_2_ and that of 0.20 nm corresponds to the (100) face of 2H-phase MoS_2_ [30,31]. Additionally, the lattice’s atomic ordering is disordered due to being made up of curled edges, which would expose more defect sites and make the deposition of nickel clusters more favorable [32]. This is further understood from the disrupted lattice stripes. The EDS mapping in Figure 1e provides more details on the sample’s consistent distribution of Mo, S, Ni, and C.

**Figure 2:**
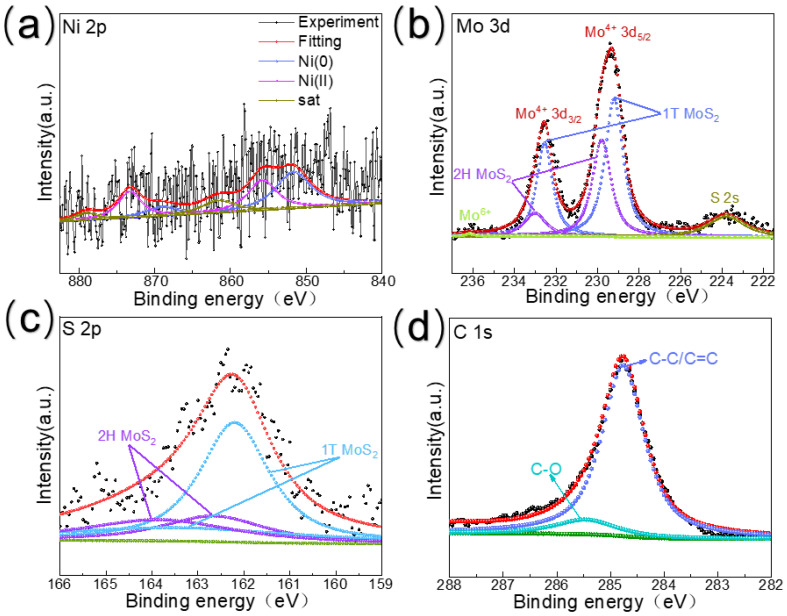
XPS spectra of MoS_2_@Ni/RGO: (**a**) Ni 2p, (**b**) Mo 3d, (**c**) S 2p, and (**d**) C 1s.

The chemical composition of the components was analyzed using XPS. The XPS spectra of Ni 2p, shown in Figure 2a, show binding energies at 851.9 eV and 869.0 eV, which correspond to Ni^0^, from which we speculate that the addition of Ni clusters may stimulate charge polarization, prompting the transfer of electrons to the Ni clusters and further increasing the number of transferable electrons, while the binding energies at 855.8 eV and 873.7 eV, which correspond to Ni^2+^, are due to the irreversible oxidation of the surface of the samples during the testing process, whereas related studies have shown that the optimization of the electron-configuration active sites may come from high-valent metal ions, which would be more favorable to the reaction kinetics [33,34]. From Figure 2b, we learn that the two well-separated peaks in the spectrum of Mo 3d correspond to the Mo 3d_5/2_ and Mo 3d_3/2_ orbitals, respectively. After processing, these two peaks are correspondingly assigned as the characteristic peaks of 1T-MoS_2_ and 2H-MoS_2_. Specifically, the 3d_5/2_ and 3d_3/2_ peaks of Mo(IV) in 1T-MoS_2_ corresponded to 229.2 eV and 232.5 eV, respectively, whereas the 3d_5/2_ and 3d_3/2_ peaks of Mo(IV) in 2H-MoS_2_ corresponded to 229.8 eV and 233.01 eV. This is in agreement with the results of the XRD data, which proved the existence of both 1T and 2H structures in the prepared MoS_2_ samples. In addition, the 236.2 eV binding energy corresponds to Mo^6+^, while the 223.8 eV binding energy corresponds to the S 2s peak. Among them, Mo^4+^ comes from molybdenum disulfide, while Mo^6+^ comes from molybdenum oxide formed by slight oxidation of the surface. It is noteworthy that the scattering angle of the characteristic peak of Mo^6+^ is much smaller than that of the characteristic peak of Mo^4+^, which further indicates that the catalyst is mainly composed of molybdenum disulfide with very little molybdenum oxide [35,36,37,38,39]. The results of the XRD and HRTEM tests corroborate the aforementioned findings, which further show the success of the synthesis of MoS_2_@Ni/RGO.

The DFT calculations of the electron density difference map and the Bader charge analysis were performed to further study the electron redistribution on the MoS_2_@Ni/RGO structure. The different types of electron density maps display that the presence of graphene results in the electron cloud on the Ni clusters becoming denser and causing the transferable charge to grow. This is additionally supported by the XPS spectra and the Bader charge analysis. The analysis makes sense. The results presented here support the redistribution of electrons in MoS_2_@Ni/RGO.

### 2.2. HER Catalytic Behavior

**Figure 3:**
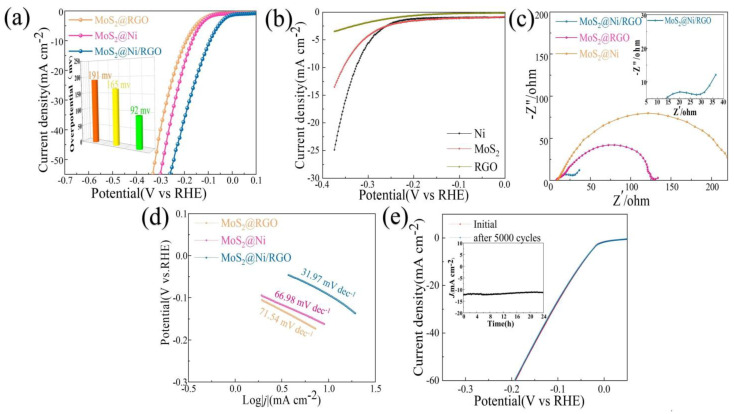
The HER behavior of MoS_2_@Ni/RGO, MoS_2_@Ni, and MoS_2_@RGO in 1 M KOH. (**a**) The LSV curves and overpotential (*η*_10_) without IR correction. (**b**) LSV curves for pure MoS_2_, pure Ni, and pure RGO. (**c**) Nyquist plots collected at the overpotential of 180 mV. (**d**) Tafel slopes. (**e**) The initial and 5000th polarization curves of MoS_2_@Ni/RGO. The inset is the chronoamperometric curve recorded at 10 mA for a continuous 24 h.

In a 1 M KOH solution, we examined the effect of the presence of MoS_2_@RGO, MoS_2_@Ni, and MoS_2_@Ni/RGO on HER. As appears in Figure 3a, HER activity for MoS_2_@Ni/RGO was strongly enhanced. The overpotential of MoS_2_@RGO at 191 mV was 10 mA·cm^−2^ (*η*_10_), which is in line with the data that have been provided in the pertinent literature. Compared with MoS_2_@Ni (165 mV) and MoS_2_@RGO (191 mV), *η*_10_ for MoS_2_@Ni/RGO was significantly reduced at 92 mV. The electrocatalyst performance mentioned in the pertinent literature is equivalent to MoS_2_@Ni/RGO’s good performance in alkaline solutions, as suggested in Figure 3a (see Table 1 for additional information). We similarly checked the HER performance of pure RGO, MoS_2_, and Ni under alkaline conditions to highlight the synergistic impact even more. The catalytic activity performance of pure RGO, MoS_2_, and Ni under alkaline conditions was not particularly impressive, as shown in Figure 3b.

**Figure 4:**
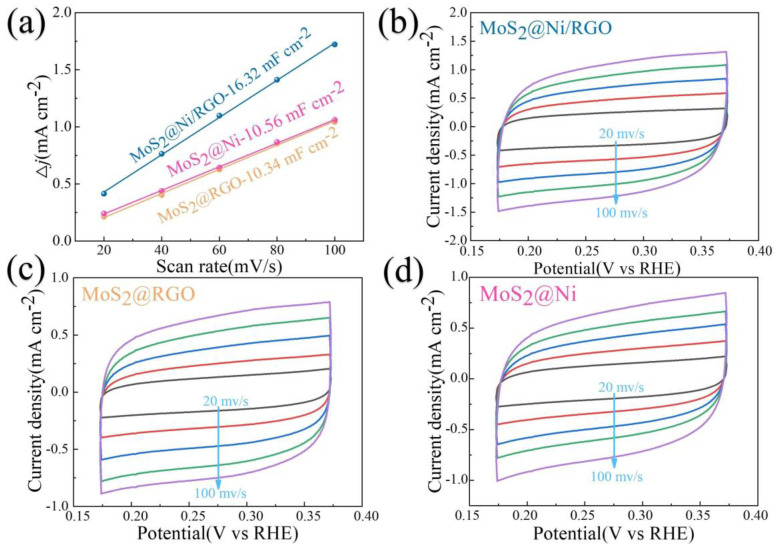
(**a**) Differences in current density variation (Δ*J = J_a_* − *J_c_*) at 0.05 V vs. RHE plotted against scan rate fitted to linear regression for estimation of Cdl values of MoS_2_@Ni/RGO, MoS_2_@Ni, and MoS2@RGO. (**b**–**d**) CV curves of MoS_2_@Ni/RGO, MoS_2_@RGO, and MoS_2_@Ni at the scan rates of 20, 40, 60, 100, and 30 mV·s^−1^, respectively.

The Tafel slope, a place that can be observed in Figure 3d as a Tafel curve, can also be used to further draw attention to the quickly occurring and gradual entry of various samples into reaction kinetics. The results from this study demonstrate that graphene is vital for improving the kinetics of the HER reaction, which is shown by the Tafel slopes of MoS_2_@Ni/RGO (31.97 mV·dec^−1^), MoS_2_@RGO (71.54 mV·dec^−1^), and MoS_2_@Ni (66.98 mV·dec^−1^). The reality that MoS_2_@Ni/RGO has the lowest charge transfer resistance among the catalysts in Figure 3c suggests that it has an advantage when determining the reaction kinetics and electrical conductivity. A vital component of the electrochemical performance index is the electrochemically active surface area (ECSA). To make this comparison more straightforward, we calculated the ECSAs of MoS_2_@Ni/RGO, MoS_2_@RGO, and MoS_2_@Ni by using CV curves. At scan rates of 20, 40, 60, 80, and 100 mV·s^−1^, the samples’ CV curves were examined in the 0.15–0.40 V range (Figure 4b–d). The calculated Cdl values for the different catalysts were: 16.32 mF cm^−2^ (MoS_2_@Ni/RGO), which was much higher than 10.34 mF cm^−2^ (MoS_2_@RGO) and 10.56 mF cm^−2^ (MoS_2_@Ni) (Figure 4a), and the catalyst performance enhancement was attributed to the introduction of graphene to increase the electrical conductivity on the one hand and to enable the active sites to be further exposed on the catalyst surface, which is thought to be beneficial for HER performance.

The MoS_2_@Ni/RGO structure was measured for 5000 continuous CV cycles in an alkaline environment at 0–0.3 V in order to gain an insight into its long-term stability. The basically unnoticeable distinctions between the two LSV curves before and after 5000 CV cycles (Figure 3e) reflect the higher catalytic stability of MoS_2_@Ni/RGO throughout the electrochemical reaction process. We looked further at the time–current curves of the catalysts to further confirm their durability, and the feedback data in the insert of Figure 3e show that the current decay had virtually no effect over a long time—24 h—which further proves their stability in an alkaline environment.

**Figure 5:**
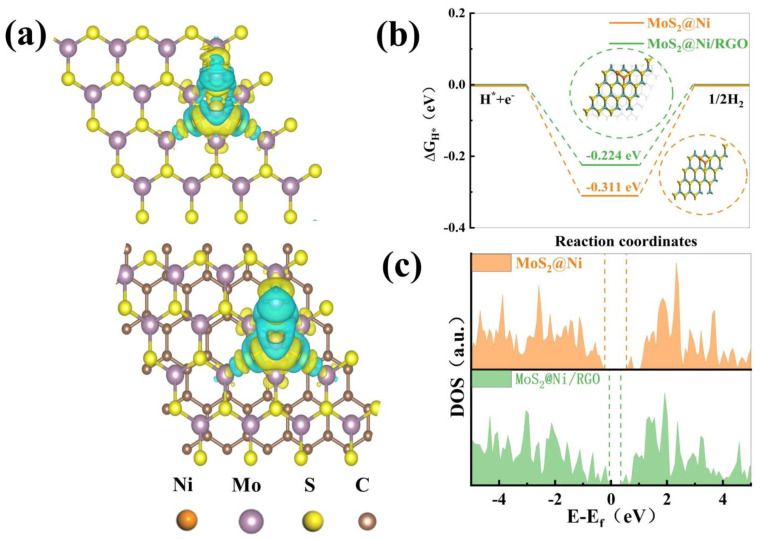
(**a**) Illustration of the electrocatalytic HER mechanism under alkaline conditions. (**b**) Free energy diagrams of MoS_2_@Ni and MoS_2_@Ni/RGO surfaces in alkaline solution. (**c**) DOS of pristine MoS_2_@Ni and MoS_2_@Ni/RGO.

It is pertinent to note, based on the information that is provided by the electronic structure, that the additional performance addition of Ni leads to the further enrichment of the active site, which in turn offers a convenient bridge for the interaction of the catalyst with the intermediate. The incorporation of graphene makes the Ni cluster electron cloud of MoS_2_@RGO denser, which also points to an increase in the number of transferable electrons, which can be observed in Figure 5a, where blue represents the loss of electrons and yellow represents the gain of electrons [40]. Hydrolytic adsorption on the catalyst, recombination of hydrogen atoms, electron transfer, and desorption to form components are the four main components of the HER reaction step in alkaline environments. Under alkaline conditions, water molecules undergo a spontaneous dissociation reaction to produce hydrogen ions (H^+^) and hydroxide ions (OH^−^). Next, when an applied potential is applied to the hydrogen electrode, hydrogen ions (H^+^) are directed to the electrode surface. At the electrode surface, the hydrogen ions accept electrons and undergo a reduction reaction to produce hydrogen gas (H_2_). However, with the addition of nickel clusters, the electronic structure and electronic states on the surface of the nickel clusters are altered, which facilitates the adsorption and dissociation of hydrogen ions and the reduction of hydrogen ions to hydrogen gas. Specifically, the electronic states of the nickel clusters can interact with the hydrogen ions, providing adsorption sites and lowering the adsorption energy barriers, thereby enhancing the ability of hydrogen ions to be adsorbed on the surface. In addition, the electronic structure of the nickel clusters can also provide additional electrons to the hydrogen ions, which further facilitates the reduction reaction of the hydrogen ions and thus enhances the production of hydrogen gas [41]. In the case of adsorption estimations of energy for H* protons, previously conducted studies shared with us that an appropriate energy barrier must be established for water adsorption/desorption and that the binding strength of hydroxide plays a crucial part in hydrogen generation in an alkaline environment. The difference that exists between the adsorption energy barriers of MoS_2_@Ni and MoS_2_@Ni/RGO in Figure 5b, −0.311 eV for MoS_2_@Ni and −0.224 eV for MoS_2_@Ni/RGO, clearly shows that the inclusion of graphene significantly improves the reaction energy barrier. The addition of graphene reduces the band gap, which is more proficient for the thermodynamic overall hydrolysis reaction, which is displayed by the density of total states (DOS) in Figure 5c [42,43,44]. The results reported here imply that good basic hydrogen precipitation based on the synergistic interaction is backed up strongly by the strong metal–carrier interaction between MoS_2_@RGO and Ni clusters.

## 3. Materials and Methods

### 3.1. Experimental Section

Maclean’s Reagent Co., Ltd., (Shanghai, China) supplied the ammonium molybdate ((NH_4_)_2_MoO_4_), ethylene glycol ((CH_2_OH)_2_), nickel(II) chloride hexahydrate (NiCl_2_·6H_2_O), sulfur powder (S), and graphene (RGO). A 5% Nefer solution was purchased from DuPont China Holdings Co., Ltd., (Shenzhen, China). All other compounds were obtained from Sinopharm Chemical Reagent Co., Ltd. (Shanghai, China), and were analytically pure. None of the compounds were further purified before usage.

#### MoS2@RGO, MoS2@Ni, and MoS2@Ni/RGO Were Synthesized

Synthesis of 1T-2H-MoS_2_: MoS_2_ was synthesized by dissolving 0.18 mmol of (NH_4_)_2_MoO_4_ in 30 mL deionized water for fifteen minutes and dissolving it into a clear solution by magnetic stirring, while adding 5 mL of (CH_2_OH)_2_ dropwise, weighing 0.2 mmol of S dissolved in the above solution and sonicating for 10 min, transferring it into a 50 mL polyvinylidene fluoride-wrapped chrome-plated autoclave, and holding it at 180 °C for 24 h.

Preparation of MoS_2_/RGO: First, a clear solution was made by dissolving 0.18 mmol of (NH_4_)_2_MoO_4_ in 30 mL of deionized water with magnetic stirring while 5 mL of (CH_2_OH)_2_ was added dropwise. Then, 0.2 mmol of S was dispersed in the aforementioned solution and 5 mg graphene was added, which was then ultrasonically processed for 10 min at 180 °C for a holding time of 24 h. The finished product was freeze-dried in a vacuum freeze dryer after being cleaned repeatedly with deionized water and ethanol.

Preparation of MoS_2_@Ni/RGO: A solution that consisted of ethylenediamine and water (1:3 (*v*/*v*)), which included 60 mg of MoS_2_@RGO, was sonicated for three hours. After the addition, stirring continued for 12 h while NiCl_2_·6H_2_O was sonicated for 30 min. After that, the product was placed in an oven to dry. The final product was heated to 450 °C and then kept there for two hours in a hydrogen (5%) and nitrogen mixture.

Synthesis of clusters of pure nickel: Thirty milliliters of ethylenediamine and water (1:3 (*v*/*v*)) were combined with 0.2 mmol of NiCl_2_·6H_2_O and ultrasonically treated for three hours at room temperature. Then, 0.05 mmol of EDTA was added and agitated continuously for 6 h in a water bath. Upon completion of the reaction, the precipitate was collected, washed with ethanol and deionized water, and then dried under vacuum in a vacuum freeze dryer, and finally held at 300 °C for two hours under a mixed atmosphere of 5% hydrogen/95% argon.

### 3.2. Characterization and Testing

Using a field emission scanning electron microscope (SEM; JSM-6701F, JEOL; accelerating voltage of 5 kV), a transmission electron microscope (TEM; JEOL-2010; operating voltage of 200 kV), and a high-resolution transmission electron microscope (HRTEM; JEOL-2010), the surface morphology and structural characteristics of the materials were analyzed. A Rigaku/Max-3A X-ray diffractometer (XRD) was used to analyze the crystal structures of MoS_2_@RGO, MoS_2_@Ni, and MoS_2_@Ni/RGO samples under Cu Kα radiation (λ = 1.54178 Å). A Mg Ka achromatic X-ray source, a tool created to examine the chemical state of the materials and process charge-corrected data with XPS Peak 4.1 software, was used to conduct X-ray photoelectron spectroscopy (XPS) tests.

A three-electrode mode was chosen at the electrochemical workstation (CHI 660E), and an aqueous potassium hydroxide (KOH) solution with a concentration of 1.0 mol/L was used for electrochemical testing. A silver/silver chloride (Ag/AgCl) electrode worked as the reference electrode, a graphite electrode served as the counter electrode, and the product of the experiment served as the working electrode. Using the Nernst equation, which translates *E*(RHE) = *E*(Ag/AgCl) + 0.059 pH + 0.197, the potential was modified to the RHE scale. Next, 5 mg of the generated catalyst, 50 µL of 5% Nafion solution, 300 µL of anhydrous ethanol, and 700 µL of deionized water were added. They were then placed in an ultrasonicator for 30 min to be sonicated. Then, the electrochemical test was carried out on the electrochemical workstation using 5µL of the solution supplied dropwise to the glassy carbon electrode. In the frequency range of 100 kHz to 0.01 Hz, electrochemical impedance spectroscopy (EIS) was carried out at an overpotential equivalent to a current density of 10 mA·cm^−2^. For the purpose of investigating cyclic stability, cyclic voltammetry (CV) curves were run between 0.1 and 0.3 V at a sweep rate of 100 mV/s. By looking into the chrono–current curves at a current density of 10 mA·cm^−2^, long-term stability was identified. All measurements were made with no IR compensation, and all electrochemical studies were performed at room temperature.

First-principles density functional theory (DFT) calculations were carried out by applying VASP software (https://www.vasp.at/, accessed on 1 April 2023). We chose a 2 × 2 × 1 k-point grid as the computational foundation for the computations and 500 eV as the energy cutoff for the work function calculations. The generalized gradient approximation (GGA), in the specific form of the model proposed by Perdew-Burke-Ernzerhof (PBE), is used for the electronic exchange correlation generalization.

## 4. Conclusions

In the final analysis, by utilizing techniques from atomic doping and interfacial engineering, we synthesized MoS_2_@Ni/RGO in the course of our study, successfully generating catalysts with an enormous boost in electrocatalytic efficiency and an immense number of active sites. We successfully synthesized MoS_2_@Ni/RGO via the advantage of interfacial electron transfer, and it exhibited a low overpotential of 92 mv, a Tafel slope of 31.97 mv·dec^−1^, and a stability of more than 20 h. The intermediate energy barrier for the dissociation of fluid can be effectively lowered by the nanocluster–carrier interactions between MoS_2_@RGO and Ni clusters, as indicated by DFT calculations. As a consequence of this, in an alkaline environment, good HER catalytic performance was observed. The work presented here offers fresh perspectives on non-precious metal doping.

## Figures and Tables

**Table 1:** Comparison of performance results reported in this work and recent work.

Catalysts	Overpotential *η* (mV)	Electrolyte	Reference
MoS_2_@Ni/RGO	*η*_10_ = 92	1.0 M KOH	This work
Ni_2_P/MoS_2_/N:CNT	*η*_10_ = 149	1.0 M KOH	[1]
Fe-MoS_2_/Ni_3_S_2_/NF	*η*_10_ = 130.6	1.0 M KOH	[2]
MoS_2_/Ni_3_S_2_	*η*_10_ = 110	1.0 M KOH	[3]
MoS_2_-Ni_3_S_2_	*η*_10_ = 98	1.0 M KOH	[4]
Ni(OH)_2_/MoS_2_	*η*_100_ = 156	1.0 M KOH	[5]
MoS_2_/NiS/MoO_3_	*η*_10_ = 95	1.0 M KOH	[6]
Ni–MoS_2_/NCNTs	*η*_10_ = 179	1.0 M KOH	[7]
NC@MoS_2_/Ni-NC	*η*_10_ = 96.3	1.0 M KOH	[8]
Ni-MoS_2_/CC	*η*_10_ = 107	1.0 M NaOH	[9]
Ni_3_S_4_-MoS_2_	*η*_10_ = 166	1.0 M KOH	[10]

## Data Availability

Not applicable.
